# Direct Cell Lysis for Single-Cell Gene Expression Profiling

**DOI:** 10.3389/fonc.2013.00274

**Published:** 2013-11-07

**Authors:** David Svec, Daniel Andersson, Milos Pekny, Robert Sjöback, Mikael Kubista, Anders Ståhlberg

**Affiliations:** ^1^Institute of Biotechnology AS CR, Prague, Czech Republic; ^2^TATAA Biocenter, Gothenburg, Sweden; ^3^Center for Brain Repair and Rehabilitation, Sahlgrenska Academy at University of Gothenburg, Gothenburg, Sweden; ^4^Sahlgrenska Cancer Center, Sahlgrenska Academy at University of Gothenburg, Gothenburg, Sweden

**Keywords:** real-time PCR, single-cell biology, single-cell gene expression, RNA spike, DNA spike, cell lysis, direct lysis, RNA purification

## Abstract

The interest to analyze single and few cell samples is rapidly increasing. Numerous extraction protocols to purify nucleic acids are available, but most of them compromise severely on yield to remove contaminants and are therefore not suitable for the analysis of samples containing small numbers of transcripts only. Here, we evaluate 17 direct cell lysis protocols for transcript yield and compatibility with downstream reverse transcription quantitative real-time PCR. Four endogenously expressed genes are assayed together with RNA and DNA spikes in the samples. We found bovine serum albumin (BSA) to be the best lysis agent, resulting in efficient cell lysis, high RNA stability, and enhanced reverse transcription efficiency. Furthermore, we found direct cell lysis with BSA superior to standard column based extraction methods, when analyzing from 1 up to 512 mammalian cells. In conclusion, direct cell lysis protocols based on BSA can be applied with most cell collection methods and are compatible with most analytical workflows to analyze single-cells as well as samples composed of small numbers of cells.

## Introduction

Gene expression profiling has traditionally been performed on rather large samples with plenty of material. However, tissues contain many cell types that respond differently to stimuli and environmental changes, which complicate interpretation. Many studies are confounded by the intrinsic heterogeneity of biological samples. With single-cell analysis this complexity is eliminated and the true response of each cell type can be studied (1, 2). Recent single-cell profiling studies have shown large variability in transcript levels among individual cells in seemingly homogeneous cell populations and have revealed previously unknown subpopulations (3, 4). Analysis of individual cells clearly opens up for new possibilities to study biological processes such as cell transitions, signaling, differentiation, and proliferation (5, 6).

Reverse transcription quantitative real-time PCR (RT-qPCR) is the golden standard for gene expression profiling (7, 8). Through the implementation of the guidelines “minimum information for publication of RT-qPCR experiments” (MIQE) the technique has become robust and reliable (9). Usually samples composed of hundreds of thousands of cells are analyzed. These samples are lysed with strong chaotropic agents that release and protect nucleic acids, which are then purified using protocols that remove contaminants and substances that might interfere with downstream RT-qPCR (10, 11). Common to these methods is that they include one or more washing steps that lead to losses. As we write, the catalog of known RNA types is growing, resulting in increased appreciation for the numerous biological functions carried out by RNA (12). A typical single-cell contains rather few transcripts of most genes. Recent RNA sequencing data suggest there are some 22,000 mRNAs in a mouse embryonic stem cell and some 505,000 mRNAs in a mouse embryonic fibroblast. The top thousand transcripts are present in 1.4–2709 molecules per stem cell and 77–7044 molecules per fibroblast (13, 14). Clearly, when analyzing single-cells any loss during extraction caused by washing can introduce serious uncertainty and even total loss of some transcripts. Hence, classical purification protocols based on washing are not suitable for single-cell analysis (15, 16). A protocol based on a lysis medium that disrupts the cell membrane, makes RNA accessible for RT and maintains RNA integrity without inhibiting the downstream enzymatic reactions offers great advantages in quantitative single-cell gene expression profiling.

In this work we study lysis buffers that are suitable for small samples (1–1000 cells) and do not require washing. We test several lysis agents in use today, comparing lysis yield, reproducibility, and RNA stability. The effect on sample handling after cell lysis is another important parameter to consider, since the time from cell collection to storage can vary from minutes to hours. We also compare the sensitivity of direct cell lysis to traditional column based RNA extraction protocols, and we test how many cells can be analyzed without downstream inhibition. To assess yields and validate reproducibilities we use RNA and DNA spikes (17).

## Materials and Methods

### Cell cultures

Primary astrocytes were generated from post-natal day 0–1 mouse brains and prepared as described (4). The astrocytes were washed twice in PBS and treated with 0.25% Trypsin/EDTA (Invitrogen) for 2 min to dissociate cells. The dissociated cells were kept on ice in either PBS supplemented with 2% BSA or in astrocyte culture medium until subsequent analysis. All experiments involving mice were conducted according to protocols approved by the Ethics Committee of the University of Gothenburg, Gothenburg, Sweden.

### RNA and DNA spikes

A Universal RNA Spike (TATAA Biocenter) was used to evaluate the performance of the lysis buffers and RT efficiencies. The RNA spike is about 1000 bases long and has a 5′ cap and a poly-A tail of approximately 200 bases to mimic eukaryotic mRNA. The spike sequence is not present in any known genome. A DNA spike was used to determine the specific effect of the lysis protocols on DNA.

### Cell lysis

Cells were sorted with a BD FACSAria (Becton Dickinson) into 96-well plates (Applied Biosystems) with 5 μl lysis buffer per well as described (2, 18). The following chemicals were evaluated (final lysis concentrations are shown): 7-deaza-2′-deoxyguanosine-5′-triphosphate lithium salt (100 μM, Sigma-Aldrich); Betaine solution (4 M, Sigma-Aldrich); BSA (1–4 mg/ml, Fermentas); guanidine thiocyanate solution (GTC) (40–80 mM, Sigma-Aldrich); GenElute linear polyacrylamide (LPA) (50 ng/μl, Sigma-Aldrich); Igepal CA-630 (also known as Non-idet P-40, 0.5–4%, Sigma-Aldrich); polyinosinic acid potassium salt (50 ng/μl, Sigma-Aldrich); RNAseOUT (10 U/μl, Invitrogen); 2× reverse transcription buffer: 100 mM Tris-HCl (pH 8.3), 150 mM KCl, and 6 mM MgCl_2_ (Invitrogen); d-(+)-trehalose dihydrate (1 M, Sigma-Aldrich); yeast tRNA (50 ng/μl, Ambion); RT mix (2× RT buffer, Invitrogen, 5 μM random hexamers (Metabion), 5 μM oligo-dT (Metabion), 1 mM dNTP); RT mix with BSA (2× RT buffer, 5 μM random hexamers, 5 μM oligo-dT, 1 mM dNTP, 1 mg/ml BSA); and RNase-free water (Gibco). The BSA (20 mg/ml) was supplied in 10 mM Tris (pH 7.4 at 25°C) 100 mM KCl, 1 mM EDTA, and 50% v/v glycerol. For detailed list of chemicals used in lysis buffer, see Table S1 in Supplementary Material. The DNA (2 × 10^5^ molecules) and RNA (2 × 10^6^ molecules) spikes were added to the lysis buffers. Lysed samples were frozen at −80°C until RT. Each lysis was tested in four replicates.

To evaluate RNA stability in different lysis buffers we performed time course studies as well as freeze-thaw cycling experiments. Briefly, after cell dissociation the concentration was adjusted to 200 cells/μl. For each test 2.5 μl cell suspension was added to 47.5 μl lysis buffer and vortexed. For the time course study, samples were kept at room temperature for 0, 1, 2, and 6 h (*n* = 4). For the freeze-thaw cycling test, lysates were frozen at −80°C for 20 min and then thawed in room temperature for 20 min. The freezing-thawing was repeated 1, 2, 3, or 6 times (*n* = 4). Two microliters of cell lysate was used for RT.

### DNA and RNA purification

Total RNA was extracted using the RNeasy Micro kit without DNase treatment (Qiagen). Cells were sorted into 75 μl RLT buffer supplied with 2-Mercaptoethanol (Sigma Aldrich), RNA and DNA spikes, and 20 ng of poly-A carrier (Qiagen) per sample. Lysed cells were frozen at −80°C until extraction, which was performed according to the manufacturer’s instructions. All purified RNA (10 μl) was used in reverse transcription. Purification of PCR products for the DNA spike was performed with Qiaquick PCR purification kit (Qiagen).

### Reverse transcription

SuperScript™ III Reverse Transcriptase (Invitrogen) was used for reverse transcription. Directly lysed cells were incubated in 0.5 mM dNTP (Sigma-Aldrich), 2.5 μM oligo-dT (Metabion), and 2.5 μM random hexamers (Metabion) at 65°C for 5 min and then chilled on ice. 50 mM Tris-HCl (pH 8.3), 75 mM KCl, 3 mM MgCl_2_, 5 mM dithiothreitol, 10 U RNaseOut, and 50 U SuperScript III were added to a final volume of 10 μl (all Invitrogen). Final RT concentrations or amounts of the agents are shown. To be able to use all RNA from the column based extraction experiments 20 μl RT reaction volumes were used in the comparison between direct lysis with 1 mg/ml BSA and columns based extraction. The temperature profile was: 25°C for 5 min, 50°C for 60 min, 55°C for 15 min followed by enzymatic inactivation by heating to 70°C for 15 min. All cDNA samples were diluted with water to 60 μl prior qPCR.

The inhibitory effect of guanidine thiocyanate in RT was tested using 32 cells comparing 10 μl RT reactions with 50 U SuperScript III to 20 μl RT reactions with 200 U Superscript III (Figure S1 in Supplementary Material).

### Quantitative real-time PCR

Quantitative real-time PCR (qPCR) was performed on the LightCycler480 (Roche Diagnostics) using SYBR Green I detection chemistry. To each reaction (10 μl) containing iQ SYBR Green Supermix (Bio-Rad) and 400 nM of each primer (Eurofins MWG Operon), 3 μl of diluted cDNA was added. Primer sequences are shown in Table S2 in Supplementary Material. The temperature protocol was 3 min at 95°C followed by 45 cycles of amplification (95°C for 20 s, 58°C for 20 s, and 72°C for 20 s). All samples were analyzed by melting curve analysis (60–95°C at 0.1°C continuous increments). Formation of PCR products of expected length was confirmed by agarose gel electrophoresis. Cycle of quantification (Cq) values were obtained by the maximum second derivative method. All biological assays were designed to span introns and were checked by the BLAST algorithm for potential pseudogenes. For *Gapdh* 308 potential pseudogenes were found. During assay validation, all primer pairs resulted in more than five cycles difference between the normal cDNA sample and the RT negative control that only contained genomic DNA. All qPCR assays were optimized to such extent that primer-dimer signals never appeared within 45 cycles of amplification, and PCR efficiencies were 90–100%. Standard curves were analyzed with GenEx (MultiD Analyses). Interplate calibrator (TATAA Biocenter) was used to compensate for instrument variation between qPCR runs. All experiments were performed according to the Minimum Information for Publication of Quantitative Real-Time PCR Experiments guidelines (9).

## Results

### Optimization of purification-free lysis

We tested the following 17 conditions for the direct cell lysis and RNA analysis by RT-qPCR in mammalian cells: water, water with RNA and DNA spikes, 100 μM 7-deaza-2′-deoxyguanosine-5′-triphosphate lithium salt (7-deaz GTP), 4 M Betaine, 1 and 2 mg/ml bovine serum albumin (BSA), 40 and 80 mM guanidine thiocyanate (GTC), 50 ng/μl GenElute LPA, 0.5 and 4% Igepal CA-630 (also known as Non-idet P-40), 50 ng/μl polyinosinic acid potassium salt (polyI), 10 U/μl RNAse OUT, 2× RT buffer, 1 M trehalose, 50 ng/μl yeast tRNA and combinations of compounds: RT mix (2× buffer, 5 μM random hexamers, 5 μM oligo-dT, and 1 mM dNTP) and RT mix + BSA (2× RT buffer, 5 μM random hexamers, 5 μM oligo-dT, 1 mM dNTP, and 1 mg/ml BSA). For details, see Table S1 in Supplementary Material. The lysis agents can be divided in groups based on function: carriers [BSA (19–21), yeast tRNA (22), LPA (23), poly I (24), and 7-deaz GTP (25)], enzymatic enhancers [BSA, betaine (25–27), trehalose (28–30)], detergent [Igepal CA-630 (1)], and chaotropic agent [GTC (1, 31)]. Most lysis conditions act through osmosis (4, 8). Each lysis protocol was evaluated on 32 primary astrocytes collected in 96-well plates using FACS (*n* = 4 for each condition). The rationale of analyzing 32 instead of single-cells in the comparison of conditions is to eliminate the effect of stochastic gene expression observed in single-cells, while keeping the number of cells still sufficiently low to reflect the lysis performance of few cells (3, 32, 33). Two highly expressed genes (*Gapdh* and *Vim*) and two intermediately expressed genes (*Dll1* and *Jag1*) were analyzed. It is common procedure to add spikes to biological samples, particularly when complex matrices are analyzed, to detect inhibition, which can strongly bias data (17, 34). To test for degradation, inhibition, and losses due to adsorption in the lysis step and downstream RT-qPCR, RNA, and DNA spikes were added to all lysis media before the cell sorting. The idea of using an RNA as well as a DNA spike is to separate the interference in RT and qPCR. The RNA spike has 3′ A-tail and 5′ Cap to mimic endogenous mRNA.

**Figure 1:**
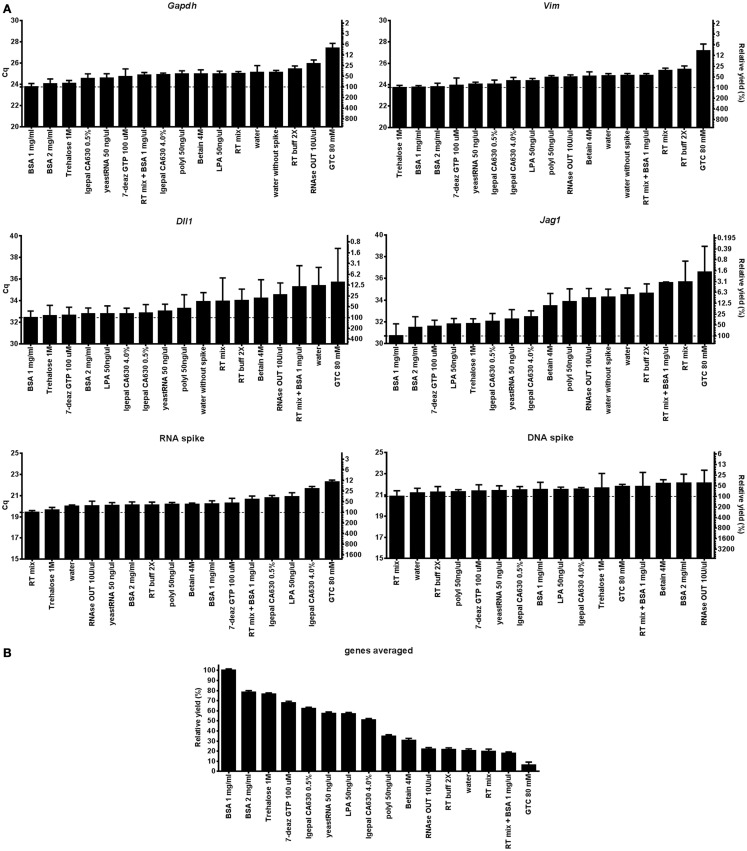
**Evaluation of direct cell lysis protocols**. **(A)** The lysis yields of *Gapdh*, *Vim*, *Dll1*, *Jag1*, DNA, and RNA spike compared at 17 lysis conditions. Thirty-two astrocytes were sorted for each condition. Relative cDNA yields are presented as Cq-values on the left *y*-axis and relative transcript numbers on the right *y*-axis. The relative transcript number is expressed in percentage compared to the optimal lysis condition for each gene, assuming 100% RT efficiency and 100% PCR efficiency. Data are shown as mean ± SD (*n* = 4). Missing data were excluded and are listed in Table S3 in Supplementary Material. **(B)** Mean cDNA yield of the transcripts. Expressions of *Gapdh*, *Vim*, *Dll*, and *Jag1* were averaged and are compared to the overall optimal lysis condition (1 mg/ml BSA). Data are shown as mean ± SD (*n* = 4). 7-deaz GTP, 7-deaza-2′ deoxyguanosine 5′ triphosphate lithium salt; GTC, guanidine thiocyanate; LPA, linear polyacrylamide; polyI, polyinosinic acid potassium salt; 2× RT buffer, 2× reverse transcription buffer; RT mix, 2× RT buffer, 5 μM random hexamers, 5 μM oligo-dT, and 1 mM dNTP.

Figure 1A shows Cq-values measured by RT-qPCR representing relative cDNA yields of *Gapdh*, *Vim*, *Dll1*, *Jag1*, and of the DNA and RNA spikes at the various tested conditions. Figure 1B shows that the use of 1 mg/ml BSA results in highest average cDNA yields for *Gapdh*, *Vim*, *Dll1*, and *Jag1*. About 2 mg/ml BSA and 1 M trehalose show almost as good behavior. The effect of lysis agent is substantial as reflected by the difference of 5.9 cycles for *Jag1* between using 1 mg/ml BSA and using 80 mM GTC. At 100% PCR efficiency this would correspond to 58-fold difference in the measured *Jag1* level. There is some variation in lysis yield with condition and also with transcript, but generally lysis was efficient with BSA. Another way to compare lysis is by the rate of positive qPCR reads for the target molecules. For the highly abundant *Gapdh* and *Vim* transcripts as well as for the two spikes all samples were positive, while for the low abundant transcripts *Dll1* and *Jag 1* the rate of positive reads ranged from 25 to 100% (Table S3 in Supplementary Material). The Cq-values measured for the DNA spike reflect the qPCR performance including inhibition and any losses due to surface adsorption in the particular matrix. There is modest variation in yields (Figure 1). Notably, RNaseOUT is the agent inducing lowest yield. For the RNA spike, which reflects the combined effect of the lysis matrix, RT, and qPCR, differences are larger. While most additives show modest variation from the RT mix, the yield dropped 7.3-fold (assuming 100% PCR efficiency) when using 80 mM GTC.

**Figure 2:**
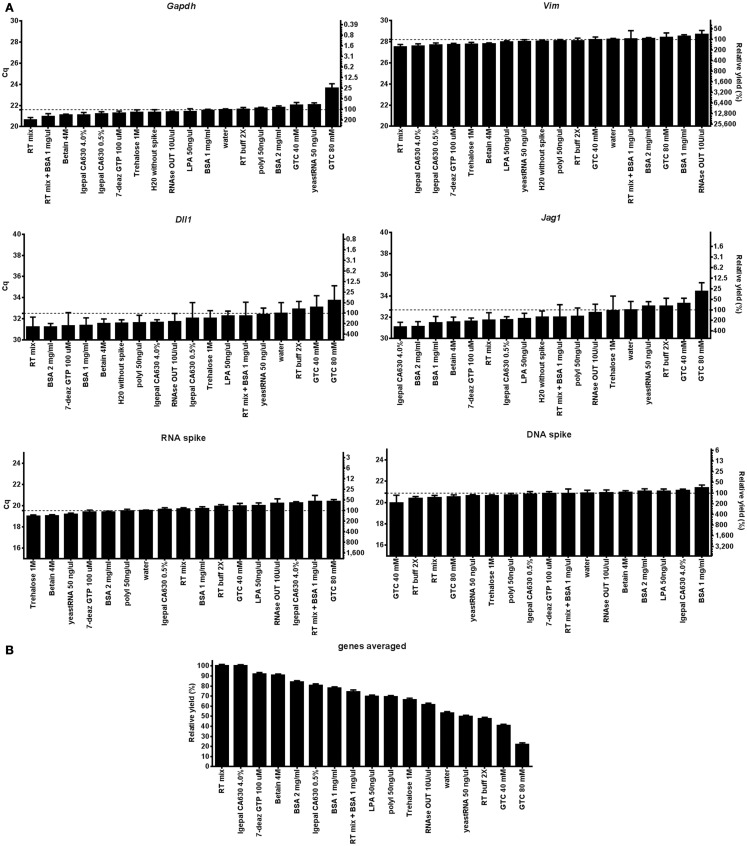
**Evaluation of direct cell lysis protocols on RT-qPCR**. **(A)** The RT-qPCR yields of *Gapdh*, *Vim*, *Dll1*, *Jag1*, DNA, and RNA spike using 17 lysis conditions. Five nanograms of purified RNA was used in all RT reactions. Relative RT yields are presented as Cq-values on the left *y*-axis and relative transcript numbers on the right *y*-axis. The relative transcript number is expressed in percentage relative to the water control for each gene, assuming 100% RT efficiency and 100% PCR efficiency. Lysis conditions with Cq-values below that of the water control are RT enhancing agents, while conditions with higher Cq-values are inhibitory. Data are shown as mean ± SD (*n* = 4). Missing data were excluded and are shown in Table S4 in Supplementary Material. **(B)** Mean RT yield for *Gapdh*, *Vim*, *Dll*, and *Jag1*. The relative transcript yield of each transcript was averaged and compared to the optimal RT-qPCR condition (RT mix). Data are shown as mean ± SD (*n* = 4). 7-deaz GTP, 7-deaza-2′ deoxyguanosine 5′ triphosphate lithium salt; GTC, guanidine thiocyanate; LPA, linear polyacrylamide; polyI, polyinosinic acid potassium salt; 2× RT buffer, 2× reverse transcription buffer; RT mix, 2× RT buffer, 5 μM random hexamers, 5 μM oligo-dT, and 1 mM dNTP.

To separate the effect of the agent added on cell lysis from that on the RT-qPCR we also analyzed 5 ng of purified total RNA from the same cells with each lysis condition. Figure 2 shows the effect of the lysis agents on the RT-qPCR only for *Gapdh*, *Vim*, *Dll1*, *Jag1*, and the RNA and DNA spikes. Most lysis agents enhance the RT yield compared to the water control (Figure 2B), exception was GTC (80 mM) which severely inhibited RT. The stimulatory effect of the lysis agents was to some degree gene (or rather assay) dependent, as can be seen by comparing the best and worst condition for *Vim*, which was 2.2-fold when comparing RT mix with 10 U/μl RNaseOut, and *Jag1*, which was 10.2-fold when comparing 4% Igepal630 with 80 mM GTC (Table S4 in Supplementary Material). The cDNA yield depends on lysis efficiency, RNA integrity, RT primer access to target RNA, and the RT yield (35). The effect of lysis agents on cell lysis together with RT-qPCR (Figure 1B) compared to RT-qPCR only (Figure 2B) does not correlate, indicating that cell lysis is the limiting step to obtain high cDNA yield.

We have previously shown that lysis of cell aggregates may be improved by addition of GTC (1). That protocol, however, requires using more reverse transcriptase. In this study we used about half the amount, which leads to the severe inhibition by GTC we observe (Figure S1 in Supplementary Material).

### Comparison of RNA stability in different lysis buffers

**Figure 3:**
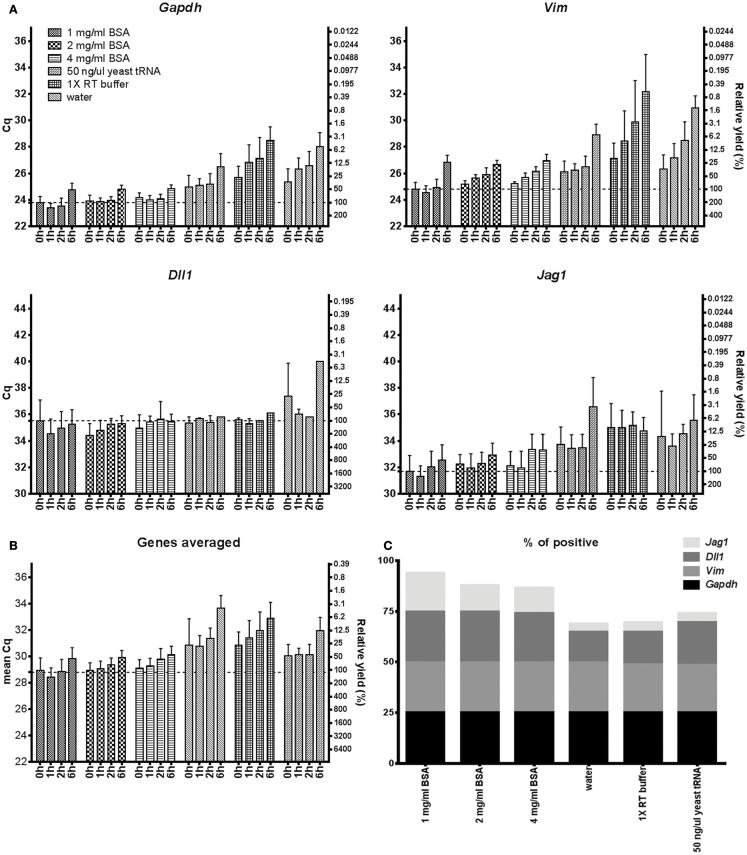
**mRNA accessibility over time**. **(A)** mRNA accessibility over time in 1–4 mg/ml BSA, 50 ng/μl yeast tRNA, 1× RT buffer, and water. Five hundred astrocytes were lysed and kept in room temperature for 0, 1, 2, and 6 h. Cq-values are shown on the left *y*-axis and relative transcript numbers on the right *y*-axis. Relative transcript number is expressed in percentage compared to the 1 mg/ml BSA sample at 0 h, assuming 100% RT efficiency and 100% PCR efficiency. Data are shown as mean ± SD (*n* = 4). **(B)** Mean RNA accessibility of the transcripts. Expression of *Gapdh*, *Vim*, *Dll*, and *Jag1* were averaged and compared to the 1 mg/ml BSA condition at 0 h. **(C)** Percentage of positive data points. Missing data were excluded from subplots **(A,B)** and are shown in Table S5 in Supplementary Material. Four genes and four time points were analyzed per lysis condition. GTC, guanidine thiocyanate; 1× RT buffer, 1× reverse transcription buffer.

RNA stability in terms of decay and accessibility after collection and cell lysis is an important but rarely tested property of lysis buffers, as the time from cell collection and lysis to analysis may vary from minutes to hours. The sample handling may also require freezing/thawing steps. We tested the stability of RNA by keeping ∼500 lysed astrocytes in six different lysis conditions at room temperature (*n* = 4) for 1, 2, and 6 h. Astrocytes that were lysed and immediately reverse transcribed were used as control. Figure 3 shows the relative stabilities of *Gapdh*, *Vim*, *Dll1*, and *Jag1* transcripts in water, 50 ng/μl yeast tRNA, 1–4 mg/ml BSA, and 1× RT buffer. The storage in BSA was superior. As expected the amount of accessible transcripts decreased with time of storage at room temperature (Tables S5 and S6 in Supplementary Material). Notably, accessible *Gapdh* and *Vim* transcript levels decreased rapidly when using lysis conditions other than BSA, while *Dll1* and *Jag1* showed more moderate decrease at all conditions. Consequently, RNA loss is gene dependent, which is in agreement with previous reports (36, 37).

**Figure 4:**
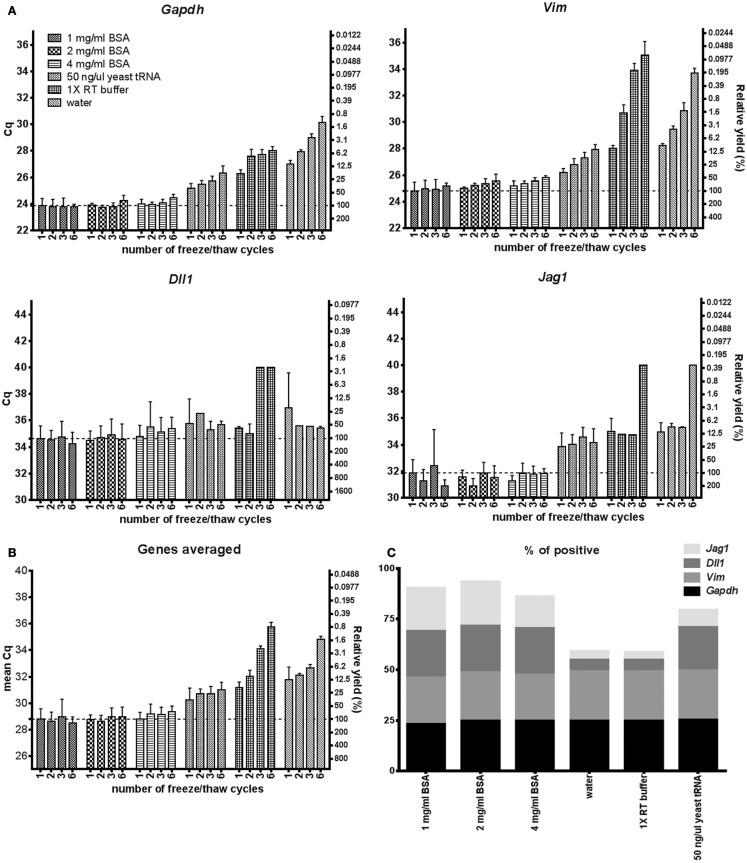
**mRNA during freeze/thaw cycling**. **(A)** Comparison of RNA accessibility after freeze/thaw cycles in 1–4 mg/ml BSA, 50 ng/μl yeast tRNA, 1× RT buffer and water. Five hundred astrocytes were lysed, frozen in −80°C and thawed in room temperature 1, 2, 3, or 6 times. Cq-values are shown on the left *y*-axis and relative transcript numbers on the right *y*-axis. Relative transcript number is expressed in percentage compared to the 1 mg/ml BSA sample thawed once, assuming 100% RT efficiency and 100% PCR efficiency. Data are shown as mean ± SD (*n* = 4) **(B)** Mean RNA accessibility of the transcripts. Expression of *Gapdh*, *Vim*, *Dll*, and *Jag1* were averaged and compared to the 1 mg/ml BSA sample thawed once. **(C)** Percentage of positive data points. Missing data were excluded from subplot **(A,B)** and are shown in Table S6 in Supplementary Material. Four genes and four different amounts of freeze/thaw cycles were analyzed per lysis condition. GTC, guanidine thiocyanate; 1× RT buffer, 1× reverse transcription buffer.

Maintaining RNA stability throughout freeze/thaw cycles is most important when handling and storing nucleic acids. Figure 4 shows that 1–4 mg/ml BSA is superior to the other tested agents to maintain RNA stability after 1, 2, 3, and 6 cycles of freezing/thawing. Using BSA in storage media almost all mRNA remains available for analysis even after six freeze/thaw cycles, while with the other agents the mRNA is gradually lost.

### Sensitivity, yield and dynamic range of RNA analysis with direct cell lysis compared to column based RNA purification protocols

**Figure 5:**
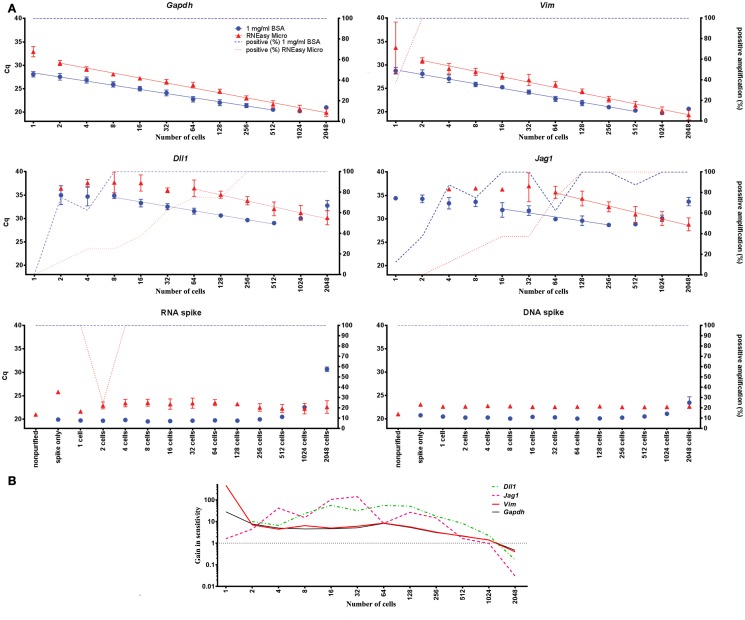
**Comparison of direct cell lysis and column based extraction**. **(A)** One to 2048 cells in steps of two were FACS-sorted and mRNA was extracted either by direct cell lysis or RNeasy Micro columns for RT-qPCR analysis. The difference in Cq-values (left *y*-axis) between 1 mg/ml BSA and column based extraction reflects the difference in sensitivity between the two methods. Percentage of samples with detectable cDNA is plotted on the right *y*-axis. The dynamic range for the endogenously expressed genes is shown by linear curve fits. The “Spike only” control sample shows the effect of column based extraction of the spikes alone without any cell material. Data are shown as mean ± SD (*n* = 4) (see also Table S7 in Supplementary Material). **(B)** Comparison of direct cell lysis to column based extraction for all transcripts. The mean difference in sensitivity is shown by averaging the expression of *Gapdh*, *Vim*, *Dll*, and *Jag1*. The dotted line, at a value of one, indicates when column based extraction is as efficient as direct lysis. Missing data were replaced with a Cq-value of 40.

To assess sensitivity, yield and dynamic range of the here optimal direct cell lysis protocol (1 mg/ml BSA) we compared it to a standard protocol based on traditional spin-columns (RNeasy Micro kit, Qiagen). Expression of *Gapdh*, *Vim*, *Dll1*, *Jag1*, and the RNA and DNA spikes were measured in FACS-sorted primary astrocytes performing a twofold dilution series ranging from a single-cell to 2048 cells (*n* = 4 per step, in total 12 steps). Cells were sorted into 5 μl of either 1 mg/ml BSA or RNeasy Micro kit RLT buffer (supplemented with poly-A carrier and 2-Mercaptoethanol). Figure 5 shows the yields and dynamic ranges of the direct cell lysis and of the column based extraction. The yields are 3- to 15-fold higher for the endogenously expressed mRNAs with the direct cell lysis protocol when analyzing 256 cells and even higher when analyzing lower cell numbers (Table S7 in Supplementary Material). For the RNA and DNA spikes the yields with direct lysis were 14.6- and 5.1-fold higher, respectively. This corresponds to more than 90% loss of RNA and 80% loss of DNA with the column based purification protocol. *Jag1* and *Dll1* transcripts were present at low levels when few cells (<16 cells) were analyzed. Here, sensitivity can be assessed from the percentage of positive replicates (samples that gave rise to a specific PCR product, Figure 5A). This comparison shows that direct cell lysis based on 1 mg/ml BSA is superior to RNeasy Micro spin-columns. The dynamic range of the direct cell lysis is indicated by linear analysis in Figure 5A. The yields of *Gapdh*, *Vim*, *Dll1*, *Jag1*, and the RNA and DNA spikes start to decline when 512 or more astrocytes were analyzed.

## Discussion

Traditional recommendations and guidelines for purification of nucleic acids refer to direct cell lysis as being inhibitory and even impossible in many conditions (11). For most sample types this is true. But for samples of low complexity, such as cell cultures and single-cells, direct cell lysis offers advantages by eliminating losses due to washing and therefore results in higher yields. Other advantages are that the protocols are simple, fast, and cost efficient, which makes them very suitable for high throughput applications (38, 39). For single-cell gene expression profiling studies, direct cell lysis is practically the only way to retrieve mRNA for reliable analysis. In fact, direct lysis can even be used for multi-analyte profiling, measuring RNA, DNA, and proteins from the same single-cells (single-cell omniomics) (40). In a recent comparison of commercial direct lysis agents with extraction using spin-columns, superior yields were obtained with direct cell lysis for up to 1000 fibroblast cells (41).

We have evaluated 17 direct lysis protocols on astrocytes comparing yield, RNA stability, and compatibility with downstream RT-qPCR. For testing of RT and qPCR proficiencies we used RNA and DNA spikes. We found best performance using 1 mg/ml of BSA. BSA is a common enhancer in PCR (21, 42). Its mechanism of action is complex and includes being a carrier (19, 43), proteinase inhibitor (44), and to sequester inhibitors (20, 45). Here, we show BSA also has advantageous properties in direct cell lysis and for maintaining RNA accessible (Figure 1). Comparing Figures 1B and 2B we conclude that the positive effect is highest when BSA is present during cell lysis. The enhancing effect of BSA on qPCR is usually thought of as a carrier effect, i.e., BSA adsorbs to the surfaces of the reaction container reducing the number of nucleic acids that bind. After BSA, yeast tRNA, which is a typical carrier molecule, performed best in the freeze/thaw cycling study (Figure 4). This suggests that BSA has some carrier properties, but other mechanisms must contribute and should be more important, since the effect of BSA on the RNA and DNA spikes was almost negligible (Figures 1 and 2). BSA is also known to reduce the effect of inhibitors in qPCR (20, 45), possibly by binding them (19, 43). However, our RNA and DNA spike data (Figure 1) suggest that inhibition is not important when small number of cells are analyzed, which is in accordance with previous observations (15). Albumin is by far the most abundant protein in the circulatory system, being present at millimolar concentrations, and it accounts for 80% of the colloid osmotic blood pressure (46). It is carrier of fatty acids in plasma and very important buffer, stabilizing the blood pH. BSA showed stabilizing effects compared to water, RT buffer, and yeast tRNA in the stability at room temperature over time and freeze/thaw cycling studies (Figures 4 and 5), which may be related to the function it has in blood. The effects of BSA and tRNA as carriers are gene dependent. Some studies suggest interactions between BSA and RNA are unlikely (47), while other suggest the affinity of nucleic acids to BSA is highly pH and ionic strength dependent (19, 43).

Another issue in RNA purification protocols is degradation by ribonucleases (RNases) (48, 49). RNase inhibitors are often added to lysis buffers or to the RT to prevent RNA degradation. In this study we used *in vitro* cultured primary astrocytes and found no improvement using RNase inhibitors (Figures 1 and 2), suggesting enzymatic degradation by RNases is not important under our conditions. This is in accordance with our previous studies of various cell types and experimental setups (1, 2, 4, 40). RNases, including endonucleases and exonucleases are all a large family of RNA degrading enzymes. In eukaryotic cells mRNAs form ribonucleoprotein complexes with compact quaternary structures, in which 3′ mRNA ends are covered by proteins or embedded in secondary structures that protects them from intracellular RNases (50). Eukaryotic cells also produce the ribonuclease inhibitor protein (51). These protection mechanisms are likely to remain after mild direct lysis. Many RNases are also secreted and the human body fluids are very rich in RNases that are very active and extremely stable, but their functions are not well known (52). Extracellular RNases are most likely washed away while preparing dissociated cells for later cell collection. We speculate that the loss of mRNAs during storage time and freeze/thaw cycling is due to self-hydrolysis of nucleic acids, aggregation, and absorption rather than to RNase activity. Self-hydrolysis can be mitigated with optimized buffer.

Cells with more rigid plasma membrane may require harsher lysis condition for complete cell disruption that need to be compatible with downstream analysis. The development of inhibition resistant mutants of Taq polymerases allows direct amplification of DNA in blood and soil samples that may prove to be very useful in single-cell analysis when stronger detergents and salts are required (53, 54). In the future we expect direct lysis of larger number of cells and perhaps even small tissue pieces to be possible (55–57).

In conclusion, we show that direct lysis of single-cells and even few cells samples can be reliably performed without losses in combination with RT-qPCR. We also show that additives such as BSA have several advantageous properties to the lysis buffers, including high lysis yield and stabilizing effect on the mRNA.

## Author Contribution

Conceived and designed experiments: David Svec and Anders Ståhlberg. Performed the experiment: David Svec, Daniel Andersson, and Anders Ståhlberg. Analyzed the data: David Svec, Daniel Andersson, Robert Sjöback, Mikael Kubista, and Anders Ståhlberg. Contributed reagents and material: Milos Pekny, Mikael Kubista, and Anders Ståhlberg. Wrote the paper: David Svec, Daniel Andersson, Milos Pekny, Robert Sjöback, Mikael Kubista, and Anders Ståhlberg.

## Conflict of Interest Statement

David Svec, Robert Sjöback, Mikael Kubista and Anders Ståhlberg declare stock ownership in TATAA Biocenter. The other co-authors declare that the research was conducted in the absence of any commercial or financial relationships that could be construed as a potential conflict of interest.

## Supplementary Material

The Supplementary Material for this article can be found online at http://www.frontiersin.org/journal/10.3389/fonc.2013.00274/abstract
