# Supplementary material for: Direct Cell Lysis for Single-Cell Gene Expression Profiling
Source: Front Oncol. 2013 Nov 7;3:274. doi: 10.3389/fonc.2013.00274 (PMC3819639; doi:10.3389/fonc.2013.00274)
Supplement: Supplementary file 1 [file 70729_Svec_DataSheet1.PDF]

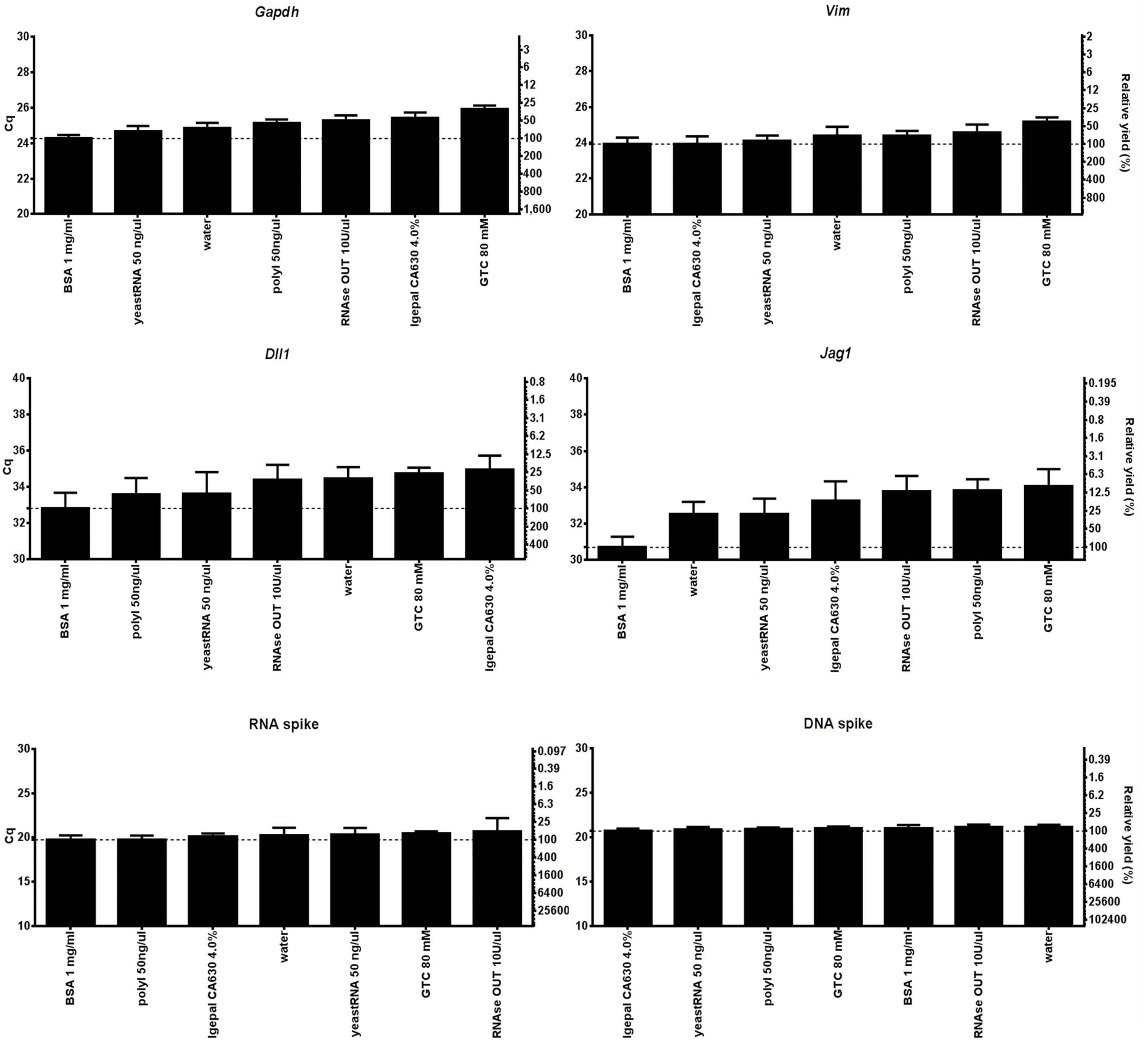

Supplementary Figure 1: Inhibitory effects of guanindine thiocyanate. Guanidine thiocyanate (GTC) showed inhibitory effects in Figures 1 and 2, while Bengtsson et al. reported that GTC can be beneficial in direct lysis (Bengtsson et al., 2008). Thirty-two astrocytes were sorted by FACS for 7 different lysis conditions. The relative cDNA yields, Cq-values on the left y-axis and relative transcript numbers on the right y-axis, using the RT-qPCR setup described by Bengtsson et al. are shown. The relative transcript number is expressed in percentage compared to the optimal lysis condition for each gene, assuming 100% RT efficiency and 100% PCR efficiency. Data are shown as mean ± SD (n=4). Missing data were excluded from the plots.

PolyI, polyinosinic acid potassium salt.
